# Serotonin and working memory in mood disorder and healthy states: multi-cohort positron emission tomography study

**DOI:** 10.1192/bjo.2026.11045

**Published:** 2026-05-25

**Authors:** Vibeke Høyrup Dam, Annette Johansen, Nic Gillings, Claus Svarer, Kamilla Woznica Miskowiak, Gitte Moos Knudsen, Dea Siggaard Stenbæk, Vibe Gedsoe Frokjaer

**Affiliations:** Neurobiology Research Unit, Copenhagen University Hospital Rigshospitalet, Copenhagen, Denmark; School of Psychological Sciences, University of Bristol, UK; Department of Neurology, Copenhagen University Hospital Rigshospitalet, Copenhagen, Denmark; Department of Clinical Physiology and Nuclear Medicine, Copenhagen University Hospital Rigshospitalet, Copenhagen, Denmark; Department of Psychology, University of Copenhagen, Denmark; Faculty of Health and Medical Sciences, University of Copenhagen, Denmark; Psychiatric Center Copenhagen, Rigshospitalet, Copenhagen, Denmark

**Keywords:** Serotonin, working memory, mood disorders, positron emission tomography

## Abstract

**Background:**

Working memory deficits are common in mood disorders and severely affect everyday functioning. Serotonin (5-HT) signalling has been implicated in depression and is also involved in cognitive functioning. However, its relevance for working memory remains largely unexplored.

**Aims:**

Using positron emission tomography (PET) brain imaging, we investigated the link between working memory and multiple 5-HT brain features in both healthy individuals and patients with mood disorders in a cross-sectional analysis of pooled data-sets.

**Method:**

We used multiple linear regression to test the associations between working memory performance and 5-HT 1B receptor (5-HT_1B_R) (healthy controls: 28), 5-HT 2A receptor (5-HT_2A_R) (healthy controls: 116), 5-HT 4 receptor (5-HT_4_R) (healthy controls: 97, patients: 98) and 5-HT transporter (5-HTT) (healthy controls: 137, patients: 12) PET binding in the frontal cortex. The frontal cortex was chosen as region of interest as it is critical for working memory functions.

**Results:**

There was no association between working memory and 5-HT_1B_R (*p* = 0.14), 5-HT_2A_R (*p* = 0.99) or 5-HTT (*p* = 0.80) frontal cortex binding in healthy controls. For the 5-HT_4_R, we observed a significant interaction effect of group status (*p* = 0.01), with patients showing a positive association (*β* = 6.51, *p* = 0.02) and healthy individuals showing no significant association (*p* = 0.16).

**Conclusions:**

We found no evidence that key 5-HT receptor systems are associated with working memory performance in healthy individuals, but did observe a positive association for 5-HT_4_R in patients with mood disorder. We speculate that although 5-HT neurotransmission markers may map onto working memory performance in the healthy state, pathologically altered 5-HT signalling may contribute to working memory deficits in mood disorders, possibly through downstream signalling and/or interactions with other neurotransmitter systems.

Working memory denotes the mental processes involved in short-term maintenance and manipulation of task-relevant information. In daily life, it can be expressed as the ability to briefly hold information in the mind and use it to solve mental tasks such as adding numbers or following a set of instructions.^1^ Importantly, many complex higher-order functions rely on intact working memory, including learning, problem-solving and decision-making.^2^ Impaired working memory can therefore severely affect everyday functioning^3,4^ and interfere with academic and professional achievements.^5,6^ Working memory deficits have been reported across a wide range of neuropsychiatric disorders,^7–9^ including major depressive disorder (MDD)^10^ where patients frequently experience difficulties even after so-called remission of the depressive episode.^11,12^ At present, we have a very limited understanding of the neurobiological mechanisms of working memory deficits in mood disorders and, consequently, no efficient clinical strategies for treating them. Functional magnetic resonance imaging studies have implicated multiple brain regions in normal working memory functioning,^13^ yet the most consistent finding is enhanced activation of the prefrontal cortex (PFC) during tasks with increasing working memory load.^14^ The importance of the PFC is further supported by lesion studies showing that frontal brain damage leads to working memory impairment in both humans^15^ and non-human primates.^16^ A prevailing theory posits that the primary function of the PFC in working memory is coordinating and regulating other brain regions involved in maintaining and manipulating task-relevant information.^14^ Notably, both structural and functional abnormalities in the PFC have reliably been shown in MDD,^17^ which could help explain the presence of working memory deficits in patients. Although molecular imaging and pharmacological intervention studies have consistently linked dopaminergic neurotransmission, and in particular the dopamine 1 (D_1_) receptor, to working memory functioning,^18^ much less is known about the potential role of other neurotransmitter systems including serotonin (5-HT) signalling. The 5-HT system consists of seven receptor families, including several subreceptors and the 5-HT transporter (5-HTT), which facilitates reuptake of 5-HT from the synaptic cleft. 5-HT is produced in the raphe nuclei in the brain stem from where serotonergic neurons project to both subcortical and cortical regions, including the PFC.^19^ Importantly, 5-HT has been strongly implicated in MDD pathology, as most standard antidepressant treatments target the 5-HT system,^20^ and recent studies have demonstrated altered 5-HT signalling in patients with MDD compared with healthy individuals.^21,22^ In addition, serotonergic neurotransmission is involved in a wide range of cognitive domains, including learning and memory functions,^23^ and recent lines of preclinical evidence also support a link to working memory functioning.^24^ Pooling data from multiple neuroimaging studies from the Cimbi database,^25^ we used positron emission tomography (PET) brain imaging to investigate the link between working memory function in both healthy individuals and patients in a depressed state and the expression of multiple 5-HT receptor systems in the frontal cortex, including 5-HT 1B receptor (5-HT_1B_R), 5-HT 2A receptor (5-HT_2A_R) and 5-HT 4 receptor (5-HT_4_R), as well as presynaptic 5-HTT levels.

## Method

### Participants

Working memory scores and neuroimaging data from 432 PET scans, including 332 scans collected from 269 healthy individuals and 100 scans collected from 92 patients with mood disorders, were available from the Cimbi database.^25^ Data were only included if time between PET scan and cognitive test date was ≤100 days. The clinical cohort consisted of patients diagnosed with MDD (*n* = 81) and seasonal affective disorder ((SAD) *n* = 11). Data was pooled across 16 independent neuroimaging studies conducted at the Neurobiology Research Unit, Copenhagen University Hospital Rigshospitalet, between 2004 and 2019, and included PET scans assessing the 5-HT_1B_R (healthy controls: 24), 5-HT_2A_R (healthy controls: 97), 5-HT_4_R (healthy controls: 89, patients: 89) and 5-HTT (healthy controls: 122, patients: 11). Inclusion criteria for all participants were age between 18 and 65 years, no significant head trauma, no significant somatic illness, no current use of psychotropic medication, fluency in Danish and no pregnancy or breastfeeding. An additional exclusion criterion for healthy individuals was history of DSM Axis I psychiatric illness. The patients with MDD and SAD were pooled from two clinical studies.^26,27^ All patients were antidepressant-free (≥2 months for MDD and ≥1 year for SAD) and met the criteria for a depressive episode (lasting <2 years) at the time of data collection; within the MDD group, 36 patients were diagnosed with first-episode depression and 45 with recurrent depression. Patients with SAD were screened with the Schedules for Clinical Assessment in Neuropsychiatry (SCAN) interview, and patients with MDD were screened with the Mini-International Neuropsychiatric Interview (M.I.N.I.); both groups had their clinical diagnoses confirmed by a trained psychiatrist.

The authors assert that all procedures contributing to this work comply with the ethical standards of the relevant national and institutional committees on human experimentation and with the Helsinki Declaration of 1975, as revised in 2013. The Cimbi database has been approved by the Danish Data Protection Agency (Capital Region protocol number: 2012-58-0004) and all data have been acquired with prior permission from the Danish Ethics Committee system and prior written informed consent from all enrolled individuals.

### Measures

#### Working memory task

Working memory was assessed with the letter-number-sequencing task from Wechsler’s Adult Intelligence Scale III (WAIS-III).^28^ In the letter-number-sequencing task, a jumbled sequence of letters and numbers is read to the participant (e.g. 2-C-3-A-1-B), who is instructed to mentally sort and recite them back to the tester in numerical and alphabetical order (i.e. 1-2-3-A-B-C). The sequences increase in length and difficulty until an upper limit for working memory capacity has been found, with a maximum score of 21 points. Cognitive testing took place in standardised test rooms and was conducted by trained neuropsychological testers.

#### PET and structural magnetic resonance imaging

Detailed descriptions of PET data acquisition are available in previous publications. Briefly, 5-HT_1B_R levels were quantified with [^11^C]AZ10419369,^29^ 5-HT_2A_R levels with [^18^F]Altanserin (*n* = 32)^30^ and [^11^C]Cimbi-36 (*n* = 65),^31,32^ 5-HT_4_R levels with [^11^C]SB207145^26,33,34^ and 5-HTT levels with [^11^C]DASB.^35,36^ PET scans were acquired on either a 3D High Resolution Research Tomography (CTI/Siemens) PET scanner ((HRRT) 388 scans) or a PET2 GE Advance scanner ((GE Advance) 90 scans) with scan time ranging between 40 and 120 min depending on radioligand scan protocol. Magnetic resonance imaging (MRI) T1-weighted images were acquired on 3T MR scanners (Magnetom Trio, Verio or Prisma; Siemens Healthcare Sector, Erlangen, Germany) and were segmented into grey matter, white matter and cerebrospinal fluid and co-registered with the PET images. PVElab (run on Matlab R2024a, Linux OpenSUSE 15.6, EU PVEout project (QLG3-CT-2000-00594), Copenhagen, Denmark, and Naples, Italy; https://nru.dk/pveout/) was used to automatically outline regions of interest from the structural MRI scan and extract PET time-activity curves for the frontal cortex.^37^

Outcome measure was defined as non-displaceable binding potential (BP_ND_) for all tracers except [^18^F]Altanserin, for which we used binding potential (BP_P_). BP_ND_ was modelled with the simplified reference tissue model for [^11^C]AZ10419369, [^11^C]Cimbi-36 and [^11^C]SB207145 scans, employing cerebellum as a reference region, and was here defined as BP_ND_ = *f*_ND_×*B*_avail_×(1/ *K*_D_), where *f*_ND_ is the free fraction of ligand in the non-displaceable tissue compartment, *K*_D_ is the dissociation constant and *B*_avail_ is the concentration of receptors available for binding.^38^ For [^11^C]DASB scans, BP_ND_ was calculated using the Ichise multilinear reference tissue method 2 (MRTM2),^39^ employing cerebellum as a reference region with the *k*_2_’ clearance rate constant for cerebellum fixed by applying the MRTM2 on time-activity curve data from a composite high-binding region (volume weighted averages of thalamus, putamen and caudate). Lastly, for [^18^F]Altanserin, scan data was acquired with a bolus injection followed by continuous infusion to produce tracer steady state in the tissue and the blood. The outcome parameter was the BP_P,_ defined as BP_P_ = *f*_P_ × *B*_avail_ /*K*_D_, where *f*_P_ is the free fraction of ligand in plasma. BP_P_ was calculated as (*C*_ROI_ – *C*_ND_)/*C*_p,_ where *C*_ROI_ and *C*_ND_ are steady-state mean count density in the region of interest and the reference region (cerebellum), respectively; *C*_P_ is the steady-state activity of non-metabolised [^18^F]Altanserin in plasma. Plasma from venous blood samples was used for the calculation of *C*_P,_ including correction for radioactive metabolites passing the blood-brain barrier.^40^ *C*_P_ was estimated using high-performance liquid chromatography as described by Pinborg et al.^41^ For a more detailed overview of scanner types and scan protocols, please see Supplementary Table 1.

### Statistical analyses

We used multiple linear regression models to assess the association between working memory performance and frontal cortex binding for each of the three 5-HT receptors and the 5-HTT. For the 5-HT_4_R and 5-HTT analyses where patient data was also available, an interaction term (clinical status by binding) was included to test whether the association between working memory performance and frontal cortex binding differed between healthy individuals and patients with mood disorders.

All models were corrected for age, gender, injected radioligand mass per kilogram and, when relevant, PET scanner type (HRRT versus GE Advance). Major Depressive Inventory score, indexing depressive symptom severity, was also included in models for patients. Additionally, for the 5-HTT model, the serotonin transporter-linked promoter region (5-HTTLPR) genotype (s-carrier versus non-s-carrier) was included as a covariate, whereas radioligand type ([^18^F]Altanserin or [^11^C]Cimbi-36) was included as a covariate in the 5-HT_2A_R model. Significance threshold was set at *α* < 0.05 and all *p*-values are reported in uncorrected form.

## Results

Table 1 provides demographic and neuroimaging characteristics of the study populations.

**Table 1:** Descriptive and tracer information about participants

Characteristic	Serotonin 1B receptor	Serotonin 2A receptor	Serotonin 4 receptor		Serotonin transporter	
	Healthy controls	Healthy controls	Healthy controls	Patients	Healthy controls	Patients
*n*	28	116	97	98	137	12
Gender, women	2 (7.1%)	47 (40.5%)	53 (54.6%)	68 (69.4%)	80 (58.4%)	7 (58.3%)
5-HTTLPR (s-carrier)	18 (66.7%)	55 (47.4%)	60 (61.9%)	66 (67.3%)	89 (65.0%)	8 (66.7%)
PET scanner (HRRT)	27 (100%)	81 (69.8%)	77 (79.4%)	98 (100%)	102 (74.4%)	12 (100%)
Age, years	32.0±10.2 [18–54]	29.3±9.3 [18–63]	28.8±9.6 [19–56]	26.6±7.7 [18–56]	25.4±7.5 [18–62]	29.9±8.4 [18–41]
Days between test and scan	3.8±15.2 [0–74]	18.2±24.6 [0–98]	19.9±24.4 [0–97]	2.8±4.5 [0–32]	14.9±22.1 [0–83]	8.5±8.7 [0–32]
BMI	25.0±4.1 [19.6–32.5]	24.1±3.4 [18.4–39.6]	23.3±2.5 [18.3–31.3]	24.1±4.9 [17.1–39.2]	23.7±3.4 [17.4–39.6]	22.3±2.7 [18.2–27.9]
LNS	12.5±2.8 [6–19]	12.7±2.5 [7–20]	13.1±2.7 [7–20]	11.8±2.8 [3–18]	12.5±2.8 [6–20]	12.2±1.5 [10–15]
Frontal cortex binding	1.4±0.3 [0.9–2.0]	^a^1.3±0.3 [0.9–2.0] ^b^1.9±0.7 [0.6–4.8]	0.6±0.1 [0.3–0.9]	0.6±0.1 [0.4–0.8]	0.4±0.09 [0.2–0.6]	0.4±0.07 [0.3–0.5]
Injected radioligand mass, mg/kg	0.02±0.01 [0.004–0.1]	0.02±0.03 [0.002–0.1]	0.03±0.03 [0.002–0.1]	0.01±0.01 [0.004–0.1]	0.04±0.04 [0.003–0.2]	0.03±0.07 [0.005–0.2]

For continuous variables, mean ± s.d. and range is provided. Frontal cortex binding values represent non-displaceable binding potential (BP_ND_), with one exception; for the serotonin 2A receptor group, two radioligand were used (a = [^11^C]Cimbi-36; b = [^18^F]Altanserin); for the [^18^F]Altanserin, binding potential (BP_p_) values are reported instead of BP_ND_. 5-HTTLPR, serotonin transporter-linked promoter region; PET, positron emission tomography; HRRT, 3D High Resolution Research Tomography PET scanner; BMI, body mass index; LNS, letter-number-sequencing task.

**Fig. 1:**
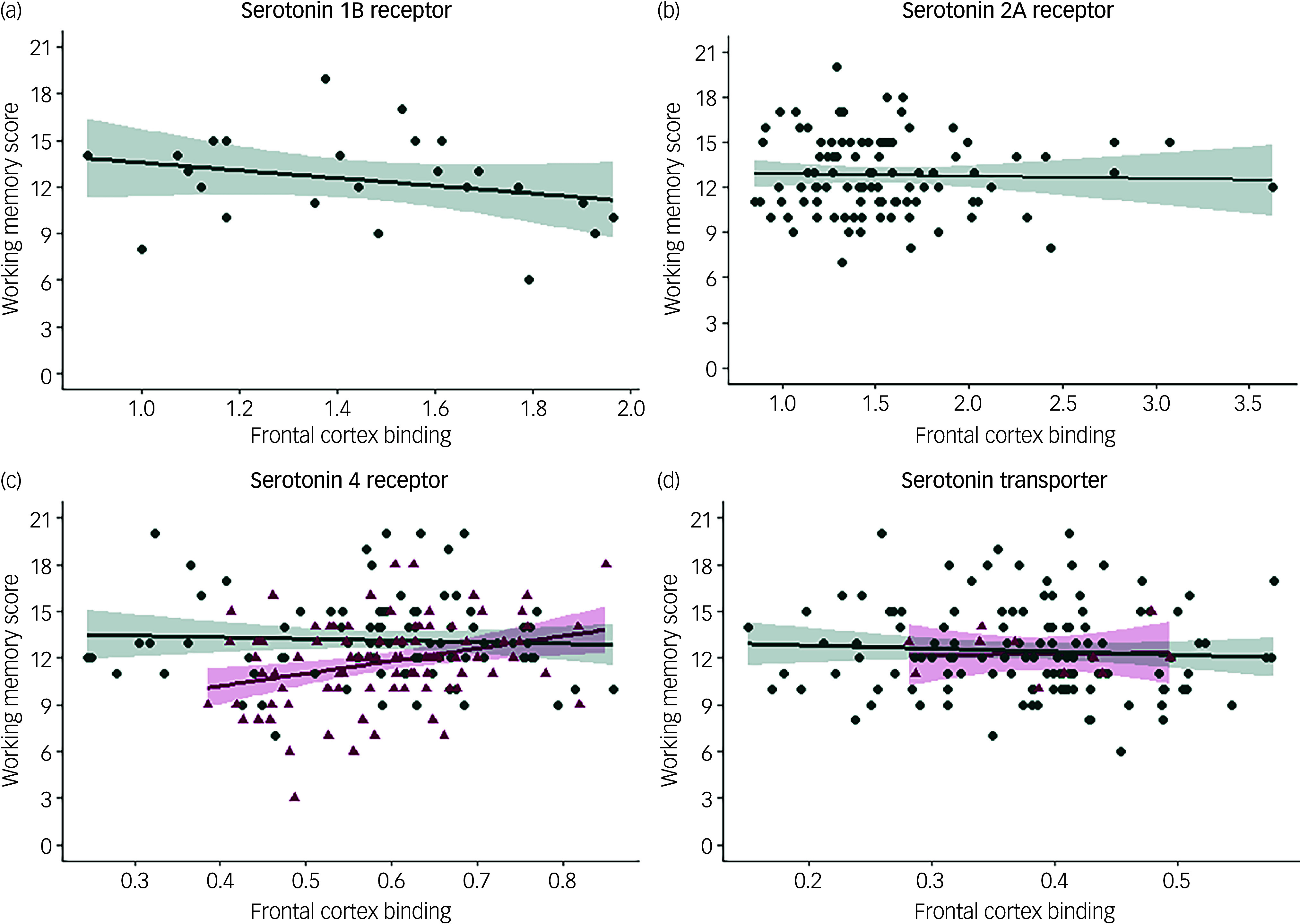
Association between working memory and serotonin brain markers. (a) Association between working memory and frontal serotonin 1B receptor binding (healthy individuals, *n* = 24). (b) Association between working memory and frontal serotonin 2A receptor binding (healthy individuals, *n* = 97). (c) Association between working memory and frontal serotonin 4 receptor binding (healthy individuals (teal circles), *n* = 89; patients (pink triangles), *n* = 89). (d) Association between working memory and frontal serotonin transporter binding (healthy individuals (teal circles), *n* = 122; patients (pink triangles), *n* = 11).

Figure 1 shows associations between working memory scores and 5-HT_1B_R, 5-HT_2A_R, 5-HT_4_R and 5-HTT frontal cortex binding.

We found no evidence of an association between working memory capacity and 5-HT_1B_R (*β* = –4.83, *p* = 0.07) or 5-HT_2A_R (*β* = 0.20, *p* = 0.83) frontal cortex binding in healthy individuals. However, there was a significant interaction effect of clinical status on the association between 5-HT_4_R binding and working memory (*p* = 0.01), with patients showing a positive association (*β* = 6.74, *p* = 0.02) and healthy individuals showing a negative but statistically non-significant association (*β* = –3.59, *p* = 0.17). As a sensitivity analysis, we repeated the analysis for patients excluding patients with an SAD diagnosis, and found that the estimates for patients with MDD only remained robust (*n* = 81, *β* = 6.42, *p* = 0.03).

We found no evidence of a group effect on the association between working memory scores and 5-HTT frontal cortex binding (*p* = 0.89), nor evidence of a main effect across groups (*β* = 1.20, *p* = 0.77). As a final sensitivity analysis, we repeated all analyses with data from participants where the time between PET scan and cognitive testing was ≤30 days (see Supplementary Table 2); the results did not differ substantially. Gender was a significant covariate in the adjusted models for 5-HT2AR (*p* = 0.002) and 5-HT4R (*p* = 0.001).

## Discussion

We report, for the first time, molecular brain imaging with PET to investigate the association between working memory capacity and multiple features of the 5-HT system in healthy individuals and patients in a depressed state. We observed a positive association between frontal cortex 5-HT_4_R binding and working memory performance in patients with mood disorders. In contrast, we found no evidence to support a direct link between serotonergic neurotransmission and working memory in healthy individuals for the 5-HT_1B_R, 5-HT_2A_R, 4-HT_4_R or 5-HTT in either healthy individuals or patients with MDD.

Until now, research into the role of 5-HT signalling in human working memory has been exceedingly sparse.^18^ The present PET cohort study is therefore not only one of the largest studies to investigate the molecular underpinning of any cognitive domain to date, but also the first to provide comprehensive insight into 5-HT contribution to human working memory.

We found no evidence that healthy-state variations in any of the 5-HT systems we investigated influence working memory performance. Yet, low 5-HT_4_R levels was associated with poorer performance in patients with mood disorders. Interestingly, the 5-HT_4_R has been highlighted as a promising therapeutic target in MDD,^42,43^ particularly for cognitive symptoms.^44^ Pharmacological activation of the 5-HT_4_R has been shown to enhance hippocampus-dependent learning and memory in rodents,^45^ whereas intervention with the 5-HT_4_R partial agonist prucalopride has produced similar memory-enhancing effects in healthy individuals.^46,47^

Further, PET neuroimaging studies have shown associations between global 5-HT_4_R binding and verbal memory performance in both healthy individuals^48,49^ and unmedicated patients with MDD,^21,50^ with the latter constituting a subgroup of the current study. We also recently reported that antidepressant-free patients with a moderate to severe depressive episode have around 7% lower cerebral 5-HT_4_R binding compared with healthy individuals,^21^ aligning with previous data showing that healthy individuals with a high familial risk of MDD also have lower striatal 5-HT_4_R binding.^51^ Although baseline 5-HT4R binding is typically interpreted as receptor availability, it has also been suggested to reflect longer term serotonergic tone,^33^ and thus group differences in MDD may reflect serotonergic dysregulation rather than solely altered receptor density. Together this indicates that altered 5-HT_4_R levels is part of depressive pathology, although it remains unclear whether low 5-HT_4_R is a driver of depressive symptoms, a compensatory mechanism or a downstream effect of other depression-related brain processes. In any case, our findings indicate that although variations in the 5-HT_4_R receptor settings are not directly linked to working memory functions in the healthy state, it becomes significant when 5-HT_4_R levels are pathologically altered. One explanation is that a still unidentified common upstream factor influences both 5-HT_4_R levels and working memory functioning. Alternatively, depression-related alterations in 5-HT_4_R signalling may modulate other neurotransmitter systems involved in working memory such as the dopamine system and thereby negatively affect working memory function. In support of this notion, Zhou et al^52^ showed that pharmacologically manipulating synaptic 5-HT levels through intervention with the selective serotonin reuptake inhibitor (SSRI) fluoxetine altered the spatial and temporal interaction between serotonergic and dopaminergic signalling in the striatum. The striatum together with the frontal cortex make up the frontostriatal circuit, which is crucial for cognitive control and adaptive manipulation of information during working memory tasks.^53^ Interestingly, two independent PET studies have found that striatal D1 receptor binding is decreased in patients with MDD.^54,55^ It is therefore plausible that striatal cross-talk between serotonergic and dopamine signalling could influence working memory in mood disorders, although future studies are needed to test this hypothesis.

Importantly, by establishing a link between 5-HT_4_R signalling and working memory function in mood disorders, our findings also highlight the potential of targeting the 5-HT_4_R as a therapeutic strategy for addressing working memory deficits in patients with mood disorders. In support of this, de Cates et al found that pharmacological stimulation of the 5-HT_4_R in healthy individuals enhanced the functional connectivity between brain networks involved in goal-oriented processes necessary for working memory.^46^ In addition, there is evidence from animal models that direct stimulation of the 5-HT_4_R may increase 5-HT tonus in the brain by stimulating 5-HT-producing neurons in the dorsal raphe nuclei, which project to the rest of the brain, including the limbic system and the cerebral cortex.^56^ Interestingly, we recently showed that 5-HT_4_R levels decrease over the course of SSRI treatment in patients with MDD, and that the patients who exhibit the largest fall in 5-HT_4_R also show the least improvement in cognitive symptoms.^50^ Together, these findings provide a strong rationale that direct 5-HT_4_R stimulation holds promise as an augmentative strategy for treating cognitive symptoms in mood disorders.

In conclusion, we provide the first evidence to suggest that 5-HT_4_R signalling may be involved in disrupted working memory function in patients with mood disorders. Our findings help point to new avenues of research into targeted pharmacological treatment of cognitive dysfunction in MDD, and highlight the importance of assessing molecular mechanisms in cognitive functions in both the healthy and pathological state, as these mechanisms appear state specific.

### Methodological considerations

Although our study is one of the largest PET studies to investigate molecular neurotransmission and cognitive functioning to date, there are several important methodological considerations that should be mentioned. First, we only looked at 5-HT_1B_R, 5-HT_2A_R, 5-HT_4_R and 5-HTT, and so did not cover all key 5-HT receptor systems that could be directly or indirectly involved in working memory functioning. Second, the healthy participant sample size for the 5-HT_1B_R and the patient sample size for the 5-HTT were both very low, and we therefore cannot exclude that we have missed a true association because of low statistical power. Third, we only had patient data for the 5-HT_4_R and 5-HTT systems; future studies are therefore needed to determine if the association between 5-HT signalling and working memory in patients with mood disorder is unique to the 5-HT_4_R or is systemic across different 5-HT receptors. Fourth, the use of different scanners and PET scan protocols across the different pooled studies may have introduced noise and obscured small effects, although this was partly mitigated by correcting for scanner type in the analysis. Finally, although SAD and MDD share core symptoms and treatment approaches, differences in underlying mechanisms mean that pooling them may introduce some variability. However, excluding patients with SAD in a sensitivity analysis had minimal impact on the 5-HT_4_R–working memory association, reducing concern about diagnostic heterogeneity. Future studies should explore diagnosis-specific effects more directly.

## Supporting information

10.1192/bjo.2026.11045.sm001 Dam et al. supplementary material Dam et al. supplementary material

## Data Availability

All study data are available upon request from the Cimbi database at Neurobiology Research Unit, Copenhagen University Hospital Rigshospitalet (https://nru.dk/).

## Supplementary material

The supplementary material is available online at https://doi.org/10.1192/bjo.2026.11045
