# Supplementary material for: Serotonin and working memory in mood disorder and healthy states: multi-cohort positron emission tomography study
Source: BJPsych Open. 2026 May 25;12(3):e146. doi: 10.1192/bjo.2026.11045 (PMC13202611; doi:10.1192/bjo.2026.11045)
Supplement: Dam et al. supplementary material [file S205647242611045Xsup001.pdf]

Supplementary Materials: Table S1 – Overview of scanner types and protocols

Target	PET tracer	MR scanner	PET scanner	PET scan protocol	
**5-HT_{1B} HC = 24**	[^{11}C]AZ10419369 HC = 24	Prisma HC = 24	HRRT HC = 24	HC = 9	3D HRRT, 120 min dynamic scan starting with AZ bolus injection, frames (listmode): 6x10 s, 6x20 s, 6x30 s, 8x2 min, and 19x5 min
				HC = 15	3D HRRT, 90 min dynamic scan starting with AZ bolus injection, frames (listmode): 6x10 s, 6x20 s, 6x30 s, 8x2 min, and 13x5 min

Target	PET tracer	MR scanner	PET scanner	PET scan protocol	
**5-HT_{2A} HC = 97**	Altanserin HC = 32	Verio HC = 5	HRRT HC = 5	HC = 12	3D HRRT, 40 min steady-state scan starting 2 hrs. after bolus injection, frames (listmode): 5x8 min, Bolus + constant infusion with Vbol=1.75
		Trio HC = 27	HRRT HC = 7		
			GE HC = 20	HC = 20	PET2 GEAdvance, 40 min steady-state scan starting 2 hrs. after bolus injection, frames: 5x8 min, Bolus + constant infusion with Vbol=1.75
	CIMBI-36 HC = 65	Verio HC = 29	HRRT HC = 65	HC = 65	3D HRRT, 120 min dynamic scan starting with Cimbi-36 bolus injection, frames (listmode): 6x10 s, 6x20 s, 6x60 s, 8x2 min, and 19x5 min
		Prisma HC= 36			

Target	PET tracer	MR scanner	PET scanner	PET scan protocol	
**5-HT_4 HC = 89 Pt = 89**	[^{11}C]SB207145 HC = 89 Pt = 89	Prisma-1 HC = 35, Pt = 81	HRRT HC = 38, Pt = 81	HC = 85 Pt = 76	3D HRRT, 120 min dynamic scan starting with SB bolus injection, frames (listmode): 6x5 s, 10x15 s, 4x30 s, 5x2 min, 5x5 min, and 8x10 min
		Verio HC = 3			

		Trio<br>HC = 51, Pt = 8	HRRT<br>HC = 38, Pt = 8		
			GE<br>HC = 13	HC = 13	PET2 GEAdvance, 120 min dynamic scan starting with SB bolus injection, frames: 6x5 s, 10x15 s, 4x30 s, 5x2 min, 5x5 min, and 8x10 min

Target	PET tracer	MR scanner	PET scanner	PET scan protocol	
**SERT<br>HC = 122<br>Pt = 11**	DASB<br>HC = 112<br>Pt = 11	Verio<br>HC = 61	HRRT<br>HC = 61	HC = 96<br>Pt = 11	3D HRRT, 90 min dynamic scan starting with DASB bolus injection, frames (listmode): 6x10 s, 3x20 s, 6x30 s, 5x1 min, 5x2 min, 8x5 min, and 3x10 min
		Trio<br>HC = 61, Pt = 11	HRRT<br>HC = 35, Pt = 11		
			GE<br>HC = 26	HC = 26	PET2 GEAdvance, 90 min dynamic scan starting with DASB bolus injection, frames: 6x10 s, 3x20 s, 6x30 s, 5x1 min, 5x2 min, 8x5 min, and 3x10 min

Table S1. Overview of scanner types and scan protocols for each of the serotonin brain targets.

Magnetic Ressonance Imaging (MRI) scanner types
Prisma. Siemens 3-Tesla Prisma scanner with a 64-channel headcoil (Siemens, Erlangen, Germany)
Verio: Siemens 3-Tesla Verio scanner with a 32-channel headcoil (Siemens, Erlangen, Germany)
Trio: Siemens Magnetom Trio 3-Tesla scanner with an 8-channel headcoil (Invivo, USA)

Positron Emission Tomography (PET) scanner types
HRRT: High-resolution research tomography Siemens PET scanner (CTI/Siemens, Knoxville, TN, USA)
GE: 18-ring GE-Advance PET scanner (General Electric, USA)

Supplementary Materials: Table S2 – Sensitivity analysis for association between working memory and serotonin brain targets with ≤30 days between cognitive test and scan dates.

Table S2. Working memory and serotonin brain targets

	N	*β*	*p*
Serotonin 1B receptor	23	-5.00	0.06
Serotonin 2A receptor	73	-0.93	0.51
Serotonin 4 receptor			
Healthy individuals	64	-5.64	0.15
Patients	88	6.68	0.02
Interaction	152	-13.06	0.008
Serotonin transporter			
Healthy individuals	97	-1.34	0.77
Patients	10	15.82	0.13
All	107	2.61	0.56
Interaction	107	6.02	0.68

Table S2. Association between serotonin brain targets indexed with positron emission tomography (PET) and working memory indexed with the Letter-Number Sequence Task (LNS). Only data from participants where scan and test date were less than 30 days apart were included. All models were corrected for age, sex, injected radioligand mass/kg and, when relevant, PET scanner type (HRRT vs GE).
